# Dietary Diversity and Its Association with Diet Quality and Health Status of European Children, Adolescents, and Adults: Results from the I.Family Study

**DOI:** 10.3390/foods12244458

**Published:** 2023-12-12

**Authors:** Marika Dello Russo, Annarita Formisano, Fabio Lauria, Wolfgang Ahrens, Leonie H. Bogl, Gabriele Eiben, Stefaan De Henauw, Antje Hebestreit, Timm Intemann, Monica Hunsberger, Lauren Lissner, Denes Molnar, Valeria Pala, Stalo Papoutsou, Alba M. Santaliestra-Pasias, Toomas Veidebaum, Maike Wolters, Alfonso Siani, Paola Russo

**Affiliations:** 1 Institute of Food Sciences, National Research Council, 83100 Avellino, Italy; marika.dellorusso@isa.cnr.it (M.D.R.); annarita.formisano@isa.cnr.it (A.F.); alfonso.siani@isa.cnr.it (A.S.); paola.russo@isa.cnr.it (P.R.); 2 Leibniz Institute for Prevention Research and Epidemiology—BIPS, 28359 Bremen, Germany; ahrens@leibniz-bips.de (W.A.); hebestr@leibniz-bips.de (A.H.); intemann@leibniz-bips.de (T.I.); wolters@leibniz-bips.de (M.W.); 3 Institute of Statistics, Faculty of Mathematics and Computer Science, Bremen University, 28359 Bremen, Germany; 4 School of Health Professions, Bern University of Applied Sciences, 3008 Bern, Switzerland; leonie-helen.bogl@bfh.ch; 5 Finnish Institute of Molecular Medicine, University of Helsinki, 00100 Helsinki, Finland; 6 Department of Public Health, School of Health Sciences, University of Skövde, 541 28 Skövde, Sweden; gabriele.eiben@his.se; 7 Department of Public Health and Community Medicine, University of Gothenburg, 405 30 Gothenburg, Sweden; 8 Department of Public Health and Primary Care, Ghent University, 9000 Ghent, Belgium; stefaan.dehenauw@ugent.be; 9 Department of Public Health and Community Medicine, Institute of Medicine, Sahlgrenska Academy, University of Gothenburg, 413 90 Gothenburg, Sweden; monica.hunsberger@gu.se (M.H.); lauren.lissner@gu.se (L.L.); 10 Department of Pediatrics, Medical School, University of Pécs, 7624 Pécs, Hungary; molnar.denes@pte.hu; 11 Department of Preventive and Predictive Medicine, Fondazione IRCCS, Istituto Nazionale dei Tumori, 20133 Milan, Italy; valeria.pala@istitutotumori.mi.it; 12 Research and Education Institute of Child Health, Attikis 8, 2027 Strovolos, Cyprus; stalo.papoutsou@gmail.com; 13 NUTRI-GENUD (Growth, Exercise, Nutrition and Development) Research Group, Faculty of Health Sciences and Sports, Instituto Agroalimentario de Aragón (IA2), Instituto de Investigación Sanitaria Aragón (IIS Aragón), Universidad de Zaragoza, 50009 Zaragoza, Spain; albasant@unizar.es; 14 CIBER Fisiopatología de la Obesidad y Nutrición (CIBERobn), Instituto de Salud Carlos III, 28029 Madrid, Spain; 15 National Institute for Health Development, Center of Health and Behavioral Science, 11619 Tallinn, Estonia; toomas.veidebaum@tai.ee

**Keywords:** diet diversity, obesity, diet quality

## Abstract

Dietary diversity (DD) plays a crucial role in fostering high-quality diets, but its association with health outcomes, particularly body adiposity and non-communicable diseases (NCDs), is inconsistent. This may be due to a lack of a standardized method for estimating DD. Our study investigates the association between two DD indices, namely the dietary diversity score (DDS) and food variety score (FVS), and anthropometric measures, biochemical parameters, and diet quality in a large population sample from the I.Family study across research centers in eight European countries. In our cross-sectional analysis of 3035 participants, DDSs varied among countries, with a higher prevalence in the third DDS tertile among those with higher education. DDS showed a positive association with diet quality across all age groups. Higher DDS tertile individuals showed increased fiber, fruit, and vegetable intake, greater meal frequency, and lower ultra-processed food consumption. No relevant biochemical differences were observed across DDS tertiles, and a higher DDS was associated with lower overweight/obesity prevalence only in adults. No significant associations were found with FVS. Our findings emphasize the need to consider food groups for a more accurate estimation of diet quality. This aligns with studies suggesting DDS alone is not an independent risk factor for obesity in children and adolescents. Public health programs should prioritize food diversity to promote improved nutrition and overall well-being in communities.

## 1. Introduction

Diet plays an important role in health promotion and disease prevention. Unhealthy eating habits (consumption of energy-dense foods, high in fats and added sugars) and sedentary lifestyles are the key factors leading to the dramatic increase in the prevalence of overweight and obesity worldwide [1]. 

Dietary guidelines are available in many countries to provide advice on what to eat and drink to prevent diet-related diseases and improve nutritional status. In the past, dietary guidelines relied essentially on the relationships between single nutrients and health outcomes. Although this approach continues, recommendations focusing on food and dietary patterns represent a more effective strategy for the prevention of non-communicable chronic diseases (NCDs) [2,3]. There is general agreement that a varied diet is necessary to ensure a healthy and adequate diet [4]. However, assessing dietary patterns is challenging and no direct methods exist. 

Among several simple proxy dietary indicators developed, dietary diversity represents a useful qualitative measure of food consumption possibly associated with healthier diets [5]. Nevertheless, there is no standardized definition of dietary diversity. In most cases, it is assessed by estimating the number of different food items or food groups consumed within a given time frame (e.g., a day, a week, or a month), using a diet assessment tool such as 24 h recalls or food-frequency questionnaires [6]. 

The different calculations and definitions of dietary diversity can indeed contribute to conflicting results in studies examining its association with health outcomes [7]. Several studies in both low- [8,9] and high-resource-setting countries [10,11,12] have demonstrated a positive association between dietary diversity and nutrient adequacy. This association suggests that individuals who consume a diverse range of foods are more likely to obtain a broader spectrum of essential nutrients, which can contribute to better overall nutritional status and health in children and adults [9,13,14,15].

Socio-economic factors are particularly important in this context, as they can impact dietary patterns [16,17,18,19]. While some studies may not fully account for socioeconomic factors, others have attempted to control for confounding variables through statistical methods or by using homogeneous study populations. Rigorous analytical approaches should be conducted to better understand the relationship between dietary diversity and children’s growth, independently of socio-economic factors [20]. 

The association between dietary diversity and NCDs exhibited inconsistent findings in previous research [21]. A recent review reported no significant link between dietary diversity and cardio-metabolic risk factors [22], while another study found a mixed association [21]. In addition, there was no consistent association observed when the outcome was adiposity or body weight [7,23]. 

A systematic review conducted in 2013 reported that dietary variety was inconsistently associated with body adiposity in diverse populations. [23]. In a subsequent review covering cross-sectional studies in adults, out of 16 studies assessed, 5 reported inverse associations, while approximately half (7 out of 16) showed no significant correlations between dietary diversity scores and body mass index (BMI). Of note, only two studies were conducted in high-income countries [7]. 

As far as we know, there is a relative lack of studies assessing the association between dietary diversity and diet quality on health outcomes for school-aged children and adolescents in high-resource countries. Several studies have demonstrated that the diets of children and adolescents are often energy dense but poor in essential micronutrients and bioactive compounds. There are several reasons for this nutritional imbalance, such as inadequate fruit and vegetable intake and a reduced dietary variety [13,16,24,25]. Regarding the association between diet diversity indices (DDIs) and obesity, the findings from studies in both children and adults have been inconsistent [24,26,27]. 

The present study aims to explore the association of two different DDIs with anthropometric indices, biochemical parameters, and diet quality in a large and well-characterized sample of European children, adolescents, and adults from the I.Family study.

## 2. Materials and Methods

### 2.1. Study Population

This cross-sectional analysis was conducted within the framework of the I.Family project (http://www.ifamilystudy.eu/ accessed on 7 September 2023), which aimed at investigating the etiology of diet- and lifestyle-related diseases in European children and adolescents. This research utilized information from the multi-center IDEFICS/I.Family cohort study, carried out across eight European countries: Belgium, Cyprus, Estonia, Germany, Hungary, Italy, Spain, and Sweden. All children aged between 2 and 10 years were eligible for inclusion, with no additional exclusion criteria applied. In total, the initial wave of the population-based IDEFICS study (2007–2008) enrolled 16,229 children from school and kindergarten settings. Among them, 11,041 also participated in a subsequent second wave (2009–2010). Furthermore, the second wave introduced 2555 newly recruited children from the same settings. The I.Family study (2013–2014) marked the third wave of the original cohort, involving a reassessment of 7118 children, along with their siblings and parents, who had taken part in either the first and/or the second wave of the IDEFICS study. Detailed information regarding the cohort has already been published [28]. 

### 2.2. Ethics

Written informed consent for all examinations was obtained from parents and children from the age of 12. Younger children were orally informed by field workers before each examination and were asked for their oral assent. Ethical approval was obtained from local institutional ethics committees at each study center. This study was conducted according to the standards of the Declaration of Helsinki and its later amendments.

### 2.3. Physical Examination

Each participant was measured for weight and height in the morning, in light clothing, and in fasting status. Weight was measured using a body composition analyzer (Tanita BC 418 MA, Tanita Europe GmbH, Sindelfingen, Germany) to the nearest 0.1 kg. Height was determined using a calibrated stadiometer (Seca 225, Seca GmbH & Co., KG., Hamburg, Germany) with an approximation of 0.1 cm. A detailed description of the anthropometric measurements in the I.Family study, including intra- and inter-observer reliability, has been published by Stomfai et al. [29]. 

BMI was calculated by dividing body weight (in kg) by height squared (in m^2^) and for all children and adolescents was transformed into age- and sex-specific z-scores calculated according to Cole and Lobstein [30]. Children and adolescents were classified as normal weight, overweight, or obese according to the cut-offs released by the International Obesity Task Force [31]. For adults, a BMI less than 25 kg/m^2^ was considered normal weight, a BMI greater than or equal to 25–29.9 kg/m^2^ was considered overweight, and a BMI greater than or equal to 30 kg/m^2^ was considered obese [31]. 

Waist circumference (WC) was measured using an inelastic tape (Seca 200, Seca GmbH & Co., KG., Hamburg, Germany), range 0–150 cm, at the midpoint between the iliac crest and the lower border of the tenth rib with the subject in a standing position with arms being relaxed on the sides, and was recorded to the nearest 0.1 cm. 

Blood pressure (BP) was assessed with an automatic device (Welch Allyn, Inc., 4200B-E2, Skaneateles Falls, NY, USA) using a cuff appropriate to the arm’s circumference. This measurement occurred after at least 5 min of rest in a seated position, following a standardized procedure [32]. Normalized (z-score) average systolic and diastolic blood pressure values were used for statistical analysis.

Fasting blood samples were collected according to standard operating procedures and levels of glucose, insulin, cholesterol, and triglycerides were measured in a central laboratory. A detailed description of blood sample collection and analytical procedures has been previously published [33]. On a subgroup, the homeostasis model assessment for insulin resistance (HOMA-IR) was calculated according to the formula: (blood glucose [mmol/L] × plasma insulin [mU/L]/22.5).

### 2.4. Socio-Demographic Data 

Socio-demographic data were collected using a questionnaire filled in at home by parents [34]. Parents self-reported their highest educational level, which was categorized based on the International Standard Classification of Education (ISCED) and classified into three main categories: low (ISCED levels 1 and 2), medium (ISCED levels 3 and 4), and high (ISCED level 5) [35]. All questionnaires were developed in English, translated into local languages, and then back-translated to check for translation errors. 

### 2.5. Dietary Data

Dietary data were collected using the web-assisted 24 h dietary recall (24-HDR), called SACANA (“Self-Administered Children, Adolescents, and Adult Nutrition Assessment”). This 24-HDR has been validated as a self-reporting instrument for assessing dietary intakes in children, adolescents, and adults [33]. A full description of the SACANA software can be found elsewhere [33]. The first 24-HDR was completed at the examination center. Participants were advised to complete another two 24-HDRs on non-consecutive days including one weekend day, over the subsequent two-week period. Participants with at least three 24-HDRs were included in the present study. Based on the individual number of recalls the mean intakes were calculated for each participant. Parents were asked to assist smaller children (<12 years) in completing their 24-HDR. Participants reported information on the amount and type of foods and drinks consumed during the previous day, starting from the first intake after waking up in the morning. Estimation of the consumed foods and beverages was supported using standardized photographs of foods in different portion sizes. Energy and nutrient intake, as well as dietary energy density, were calculated based on the German food composition table, aiming for a cross-country comparison [36]. Each food and beverage reported in the 24-HDR interview was classified according to the NOVA classification [37] and the ultra-processed food (UPF) group was considered in the analysis on the basis of the extent and purpose of industrial food processing. The relative contribution of UPFs to the total energy intake for each participant was computed and divided into age- and sex-specific quintiles. A detailed description of the UPF calculation can be found in Lauria et al. [38].

The energy density of a diet was calculated as total energy intake (kcal per day) divided by the total weight of daily food intake (g per day), including solid foods only.

To evaluate dietary behavior, a food frequency questionnaire (FFQ) was used, which was part of the Children’s Eating Habits Questionnaire (CEHQ), a reproducible screening instrument. It included 43 pan-European food items clustered into 14 food groups according to their nutritional profiles [33]. The FFQ allowed us to calculate the healthy dietary adherence score (HDAS), a measure of the degree of adherence to the dietary guidelines developed according to the principles reviewed by Waijers et al. [39]. The final HDAS added up to a maximum score of 50, where the highest score indicates the highest possible adherence to the dietary guidelines [40]. We also calculated propensities to consume foods rich in sugar or fat by dividing the weekly frequency of those foods by the total frequency of all foods assessed, reflecting the relative sugar and fat intake [41] and the weekly frequency of “junk foods” defined according to Milani et al. [42].

### 2.6. Diet Diversity Indices: Dietary Diversity Score (DDS) and Food Variety Score (FVS)

The dietary diversity score (DDS) was calculated starting from the method originally developed by Kant et al. [6]. This procedure was based on five food groups (cereals, dairy products, vegetables, fruits, and protein-rich foods such as legumes, meat, fish, and eggs), according to the dietary guidelines in the WHO European Region [43]. Condiments and sauces were not considered because in SACANA they were often part of the meal, making it difficult to include those ingredients in a specific group. Energy-dense and nutrient-poor food groups, comprising alcohol, sugared drinks, sweet and salty snacks, cakes, sugar, and confectionery were also eliminated as they are considered voluptuary foods and do not belong to the aforementioned food groups. The DDS was computed as follows: for each age group and every food group, the total count of different foods consumed by all participants was determined (Total study_items); then for each participant and for each food group, the count of different foods consumed over the 3 recall days (individual items) was divided by the “total study_items” by all participants. 

The age-specific quartiles of these ratios were calculated, and the following scores were assigned: first quartile = 1.5 points; second quartile = 3 points; third quartile = 4.5 points; fourth quartile = 6 points (FG_DDS). For each participant, the scores of the different food groups were summed. The final DDS added up to a maximum of 30 points. An example of DDS calculation is shown in Table 1. A revised version of the food variety score (FVS) [44] was calculated as the ratio between the total number of different foods (individual items) and the total number of foods (total individual_items) consumed by the participant over the first 3 recall days. Table 2 shows an example of FVS calculation. 

### 2.7. Statistical Analysis

All of the analyses were performed by age groups (6- < 12 years, 12- < 20 years, ≥20 years) [45]. Participants were categorized based on DDS and FVS tertile cut-off points. The characteristics of the population were described as mean and standard deviation (SD) and categorical variables as counts and percentages (%). One-way analysis of variance (general linear model) and multiple comparisons with the Bonferroni correction per outcome and per DDI (*p*-value of 0.05) were performed to assess the diet quality and health status across the tertiles of DDS and FVS. For this purpose, the marginal means procedure was applied using the R package ‘emmeans’. Additionally, marginal unadjusted means and corresponding 95% confidence intervals (95% CI) were calculated. Binary logistic regression analysis was performed to estimate the odds ratio (OR) and corresponding 95% CI of obesity according to DDS and FVS tertiles. The analysis was adjusted for covariates: sex, age, country of origin, family ISCED, and total daily energy intake. R statistical software (R version 4.3.0) was used for the statistical analyses. 

## 3. Results

### 3.1. Characteristics of the Population

A total of 3035 participants (males 41%, normal weight 67.7%, high ISCED 64.8%), with sociodemographic and anthropometric information available, and at least three 24 h dietary recall completed, were included in the present analysis, after the exclusion of 10,307 participants (males 45.2%, normal weight 54.7%, high ISCED 48.0%) with missing data on key variables. Compared to the original full cohort, our sample is somewhat biased toward better-educated families. 

The population characteristics by DDS and FVS tertiles for each age group, are summarized in Table 3, Table 4 and Table 5. The mean DDS among study participants was 20.5 (SD, 5.1) for the 6- < 12 years age group, 21.1 (SD, 5.1) for the 12- < 20 years age group, and 20.5 (SD, 5.1) for adults. The mean FVS was 0.7 (SD, 0.1) for the younger age group, and 0.7 (SD, 0.1) for the other age groups. In both children and adults, the mean age was higher in the highest DDS tertile. Differences between DDS and FVS were found by country. For all age groups, the higher prevalence of participants from Germany was in the low DDS tertile (6- < 12 years: 20, 8.9, 6.9%; 12- < 20 years: 25, 11, 11%; ≥20 years: 26, 14, 6.5%; low, medium, and high DDS, respectively), while for participants from Sweden the higher prevalence was in the upper DDS category. Considering the levels of ISCED, in the low level we noted a lower prevalence of participants in the higher DDS tertile and an inverse situation in the high level, in all participants. No differences in BMI and waist z-score among DDS tertiles were found in the younger age group, while in teens and adults we observed lower values of BMI, BMI z-score, waist, and waist z-score in the high DDS tertile. When we considered the BMI categories, in all age groups, we noted a higher prevalence of normal weight in the high DDS tertile (6- < 12 years: 79, 77, 81%; 12- < 20 years: 75, 77, 83%; ≥20 years: 48, 51, 61%; low, medium, and high DDS, respectively). No clear trend was observed in the BMI categories across the FVS tertiles, in all age groups. 

In the low DDS tertile, DDS_cereal is the food group that contributed the most to the total score, while DDS_fruit and DDS_vegetables made the smallest contributions across all participants. In the highest DDS tertile, the DDS_fruit score is nearly four times higher compared to the lower category, and the DDS_vegetables score is twice as high. As expected, in the upper DDS tertile, the individual scores of the five food groups were homogenous. 

**Figure 1:**
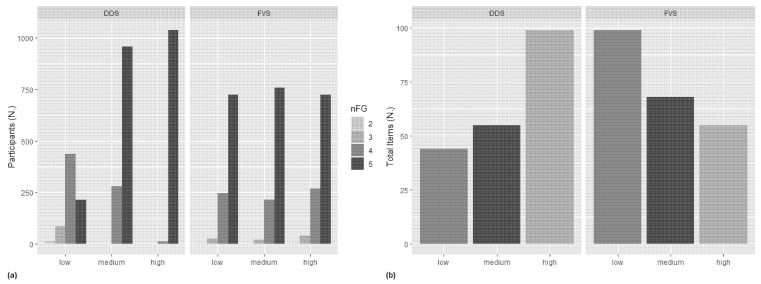
Numbers of different food groups (**a**) and foods (**b**) consumed by participants across DDS and FVS tertiles. DDS, diet diversity score; FVS, food variety score; nFG, number of food groups consumed by participants; Total Items, number of different foods consumed by participants.

We also examined the differences between the two different diet variety scores. As shown in Figure 1, while across the tertiles of DDS, there was an increase in the number of food groups consumed, in FVS the number of food groups remained unchanged across the tertiles (Figure 1a). Referring to the different foods consumed, the total number increased across the DDS tertiles, while it decreased across the FVS tertiles (Figure 1b).

### 3.2. Diet Quality and Nutritional Content According to DDS and FVS Tertiles and Age Groups

The nutritional content of consumed foods, weighted by relative intake in grams, and diet quality across the DDS and FVS tertiles by age groups are shown in Table 6. Energy intake increased across the DDS tertiles in each age group, while it was significantly low in the high FVS tertile. In the highest DDS tertile, the fat consumption expressed in percentage of the total energy intake was significantly higher in all age groups compared to the other tertiles. Saturated fatty acid (SFA) intake in percent of energy was higher in the high DDS tertile in children and teens, while the opposite situation was observed for the FVS. Carbohydrate intake, in percentage of the total energy intake, was significantly higher in the low DDS tertile in children and teens, while the opposite situation was found when referring to FVS, where energy percent intake from carbohydrates was lower in the low FVS tertile in adults. No differences in percentage contribution from protein and sugar to the total energy intake were found in all age groups. Fiber intake increased significantly across the DDS tertiles in all age groups, while the opposite was the case for FVS tertiles. The energy contribution from UPFs was high in the low DDS tertile with significant differences in teens and adults. Energy density was higher in the lower DDS category, with statistical significance in the younger age group. Considering the diet quality, HDAS, meal frequency, fruit and vegetable consumption, and fiber-rich foods were significantly lower in the first DDS tertile as compared to the others, in all age groups. The opposite was found for junk food consumption, in all age groups. When FVS was taken into account, only meal frequency was statistically significantly higher in the first FVS tertile than in the others, in adults. 

### 3.3. Health Status According to DDS and FVS Tertiles and Age Groups

Table 7 shows the association of DDS and FVS tertiles with parameters of health status by age group. At univariate analysis, there was no statistically significant difference in anthropometric parameters, blood pressure, and blood parameters across the DDS and FVS tertiles. Only in adults, we found significantly higher values of triglycerides in the lower DDS category compared to the upper.

### 3.4. Logistic Regression Analysis of the Association between DDS and FVS and BMI Categories

**Figure 2:**
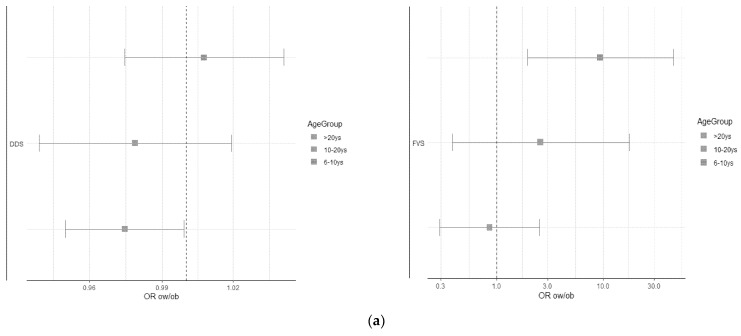

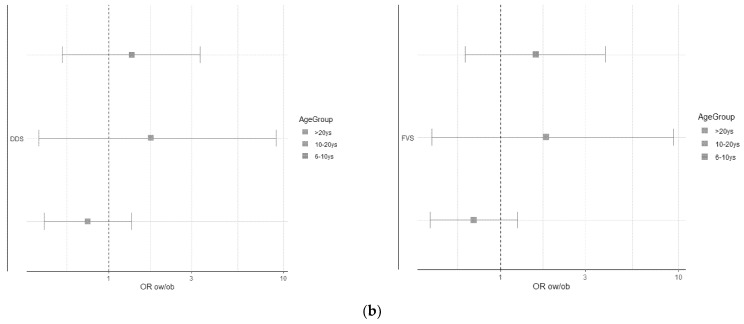
Results of the logistic regression model with BMI categories as a dependent variable. (**a**) Analysis adjusted for sex, age, country, and family ISCED (International Standard Classification of Education); (**b**) analysis adjusted for the same variables as in Figure 2a plus energy intake (kcal/day).

At binary regression analysis adjusted for sex, age, country, and family ISCED, a statistically significant association was observed between the OR of overweight/obesity and DDS in adults, as well as FVS in children (Figure 2a). However, after further adjustment for energy intake, no statistically significant association remains in children, adolescents, and adults (Figure 2b).

## 4. Discussion

In the present paper, we explored the association between two different diet diversity indices with dietary and health status parameters in a wide European population of children, adolescents, and adults, also considering participants’ sociodemographic characteristics. 

Enhancing the accessibility and variety of foods is one of the greatest global challenges. This underscores the fundamental notion that dietary diversity plays an important role in promoting well-being because it can help tackle nutrient inadequacies and improve overall nutritional status [46]. Nevertheless, there is still no consensus on the most appropriate method for estimating dietary diversity. Our findings suggest that the choice of different indicators can significantly affect the results and their interpretation. In our population, DDS was significantly and positively associated with diet quality in children, adolescents, and adults. However, marginal associations were found when considering FVS. This suggests that integrating food groups into dietary diversity assessment can provide a more comprehensive and accurate picture of nutrient adequacy compared to solely considering individual foods.

As suggested in our analysis, the dietary diversity score could be considered a rapid and efficient method for evaluating dietary and nutritional assessment, especially in large populations. In our study population, consisting of 3035 individuals from eight European countries, we found a positive association between DDS and improved diet quality. Across all age groups, the highest DDS tertile showed a statistically significant higher fiber intake, fruit and vegetable intake, as well as lower ultra-processed foods consumption. Additionally, our finding that higher DDS was associated with higher HDAS and meal frequency is consistent with previous research on adolescents and adults [47,48,49]. The positive association between dietary diversity and meal frequency could be explained, at least in part, by the fact that consuming more meals per day increases the opportunities to include a wider variety of foods per se.

The opposite happened when FVS was considered. In all age groups, energy intake was significantly higher in the low FVS tertile. The same trend was also observed for meal frequency, i.e., a higher meal frequency in the low FVS. This may be due to the fact that in our population, higher FVS did not necessarily indicate consuming a greater variety of foods from all the food groups recommended for a healthy diet. Instead, it appears to be associated with a lower number of foods consumed, as shown in Figure 1. 

The observation that individuals consuming a more varied diet had a higher energy intake but no statistically significant difference in BMI and waist circumference suggests that greater dietary diversity is associated with a healthier diet and increased consumption of low-energy-dense foods, such as fruits and vegetables. These results are in line with those of Hebestreit et al., which showed that the total number of food items consumed per day was negatively associated with energy-dense diets in children [50], and with the concept that dietary diversity may be an indicator of a more balanced and nutrient-rich diet. Our findings highlight that variety within selected food groups, rather than diversity in the whole diet per se, might have a significant impact on health promotion, as already suggested by Mirmiran et al. [13].

The lack of significant associations between dietary diversity and health status parameters or overweight/obesity risk in children and adolescents suggests that other factors beyond dietary diversity may be involved in determining health outcomes. Recent reviews and studies have reported inconsistent findings regarding the association between DDS and cardio-metabolic risk factors [7,21,22,23]. These inconsistencies may be attributed to various factors, including differences in study population characteristics, dietary assessment methods, and the composition of the DDS. To comprehensively cover global dietary intake and enhance the assessment of dietary diversity, there is a clear need for the development of new, validated DDS tools that take into account the complexities of dietary patterns and nutritional adequacy [7]. 

This study has some limitations. The cross-sectional design does not allow for the establishment of any casual associations between DDS and health outcomes including BMI status and hence a prospective association remains to be identified. Furthermore, in our analysis, we were unable to include the food group of fats in assessing DDIs, even though it is suggested to be beneficial for health, due to the constraints of the SACANA structure, which could have introduced errors. Finally, in our analysis, we did not considesr physical activity to avoid a substantial reduction in the sample size.

Despite these limitations, the study presents significant strengths. Potential confounders, such as energy intake and socioeconomic status were considered, as recommended by previous reports [22]. The present analysis examined three days of dietary intake data, thus increasing the representativeness of usual intake variations and potentially enhancing the ability to capture dietary diversity. Using standardized and validated measurements in a relatively large sample was another strength. All measurements were conducted according to standard operating procedures [33]. Furthermore, we used categorized dietary exposures which might additionally reduce bias in effect estimates [51], allowing us to take a more realistic view of the cohort’s consumption habits.

## 5. Conclusions

In summary, our findings provide valuable insights into the interplay among two different indices of dietary variety, diet quality, and health status. The study reveals that the DDS, which considers food groups, offers a more accurate estimate of diet quality compared to the FVS, which focuses only on individual foods. This highlights the importance of considering the broader spectrum of nutrients provided by different food groups. Consistent with several studies, our results indicate that DDS is not an independent risk factor for obesity in children and adolescents. Additionally, in our sample, no associations were observed between both DDIs and biochemical parameters. While acknowledging the importance of dietary diversity for nutrient adequacy and overall diet quality, it is crucial to recognize that it is just one component of a complex interplay of factors contributing to obesity risk. In light of these findings, health promotion programs should advocate for the consumption of a varied diet while emphasizing the importance of staying within calorie requirements. 

## Figures and Tables

**Table 1:** Example of DDS calculation.

ID	Age Group	FG	Individual Items (No.)	Individual Items/Total Study Items	Score FG_DDS	DDS	DDS Tertile
xxx	≥20 yrs	Cereal	5	0.0227	4.5	16.5	Low
		Dairy	2	0.0116	3.0		
		Fruit	1	0.0084	3.0		
		Protein foods	5	0.0110	4.5		
		Vegetables	2	0.0051	1.5		

ID, identification code of participant; FG, food group; Score FG_DDS, quartile of the ratio of individual items/total study items; individual items, number of different foods consumed by participants; total study_items, total number of different foods consumed by all participants in the same age group; DDS, diet diversity score.

**Table 2:** Example of FVS calculation.

ID	Age Group	Individual Items (No.)	Total Individual Items	FVS	FVS Tertile
xxx	≥20 yrs	15	17	0.88	High

ID, identification code of participant; individual items, number of different foods consumed by subject; total individual_items, the total number of foods consumed by subject; FVS, food variety score.

**Table 3:** Characteristics of participants across DDS and FVS tertiles, age group 6- < 12 years.

6- < 12 Years	All	DDS			FVS		
		Low	Medium	High	Low	Medium	High
**No.**	1017	256	426	335	342	346	329
**Sex**							
**female**	484 (48%)	115 (45%)	212 (50%)	157 (47%)	158 (46%)	163 (47%)	163 (50%)
**male**	533 (52%)	141 (55%)	214 (50%)	178 (53%)	184 (54%)	183 (53%)	166 (50%)
**Age (years)**	9.9 (1.2)	10.0 (1.3)	9.9 (1.2)	9.7 (1.2)	9.8 (1.3)	9.9 (1.3)	9.9 (1.2)
**Countries**							
**belgium**	146 (14%)	38 (15%)	76 (18%)	32 (10%)	61 (18%)	47 (14%)	38 (12%)
**cyprus**	72 (7%)	15 (6%)	32 (8%)	25 (8%)	27 (8%)	30 (9%)	15 (5%)
**spain **	74 (7%)	8 (3%)	22 (5%)	44 (13%)	15 (4%)	36 (10%)	23 (7%)
**estonia**	290 (29%)	72 (28%)	130 (31%)	88 (26%)	65 (19%)	90 (26%)	135 (41%)
**germany **	111 (11%)	50 (20%)	38 (9%)	23 (7%)	35 (10%)	34 (10%)	42 (13%)
**hungary **	106 (10%)	25 (10%)	41 (10%)	40 (12%)	33 (10%)	31 (9%)	42 (13%)
**italy **	122 (12%)	39 (15%)	59 (14%)	24 (7%)	51 (15%)	48 (14%)	23 (7%)
**sweden**	96 (9%)	9 (4%)	28 (7%)	59 (18%)	55 (16%)	30 (9%)	11 (3%)
**ISCED**							
**low**	28 (3%)	16 (7%)	11 (3%)	1 (0.3%)	10 (3%)	13 (4%)	5 (2%)
**medium**	334 (34%)	109 (44%)	132 (32%)	93 (29%)	108 (33%)	108 (32%)	118 (37%)
**high**	624 (63%)	121 (49%)	273 (66%)	230 (71%)	212 (64%)	213 (64%)	199 (62%)
**BMI z-score**	0.37 (1.07)	0.35 (1.09)	0.38 (1.07)	0.36 (1.05)	0.30 (1.06)	0.34 (1.06)	0.46 (1.08)
**Waist z-score**	0.61 (1.19)	0.58 (1.27)	0.62 (1.17)	0.60 (1.15)	0.59 (1.21)	0.55 (1.15)	0.68 (1.21)
**BMI categories**							
**Normal weight**	802 (79%)	202 (79%)	329 (77%)	271 (81%)	277 (81%)	283 (82%)	242 (74%)
**Overweight**	53 (5.2%)	11 (4.3%)	23 (5.4%)	19 (5.7%)	16 (4.7%)	14 (4.0%)	23 (7.0%)
**Obesity **	162 (16%)	43 (17%)	74 (17%)	45 (13%)	49 (14%)	49 (14%)	64 (19%)
**DDS**	20.51 (5.09)	13.59 (2.64)	20.37 (1.67)	25.97 (1.80)	19.75 (5.23)	21.36 (4.78)	20.41 (5.14)
**DDS_Cereal**	4.35 (1.39)	3.62 (1.41)	4.27 (1.35)	5.02 (1.08)	4.09 (1.42)	4.48 (1.38)	4.50 (1.34)
**DDS_ Dairy**	3.82 (1.75)	2.60 (1.58)	3.66 (1.61)	4.95 (1.28)	3.75 (1.68)	3.99 (1.68)	3.70 (1.88)
**DDS_ Fruit**	4.10 (2.39)	1.51 (2.29)	4.53 (2.03)	5.55 (0.85)	3.95 (2.45)	4.38 (2.23)	3.97 (2.48)
**DDS_ Protein foods**	4.11 (1.58)	3.01 (1.35)	3.91 (1.43)	5.19 (1.19)	4.02 (1.56)	4.26 (1.56)	4.03 (1.60)
**DDS_ Vegetables**	4.13 (1.66)	2.86 (1.52)	4.00 (1.49)	5.26 (1.14)	3.93 (1.65)	4.24 (1.65)	4.20 (1.68)
**FVS**	0.67 (0.11)	0.66 (0.14)	0.68 (0.10)	0.68	0.56 (0.06)	0.67 (0.03)	0.79 (0.06)

Values are expressed as mean (SD) for continuous variables and as counts (%) for categorical variables. ISCED, International Standard Classification of Education. DDS, diet diversity score. FVS, food variety score.

**Table 4:** Characteristics of participants across DDS and FVS tertiles, age group 12- < 20 years.

12- < 20 Years	All	DDS			FVS		
		Low	Medium	High	Low	Medium	High
**No.**	666	150	262	254	212	219	235
**Sex**							
**female**	366 (55%)	72 (48%)	152 (58%)	142 (56%)	111 (52%)	121 (55%)	134 (57%)
**male**	300 (45%)	78 (52%)	110 (42%)	112 (44%)	101 (48%)	98 (45%)	101 (43%)
**Age (years)**	13.6 (1.0)	13.6 (1.0)	13.5 (0.8)	13.6 (1.1)	13.5 (1.0)	13.5 (1.0)	13.7 (0.9)
**Countries**							
**belgium**	50 (7.5%)	13 (8.7%)	23 (8.8%)	14 (5.5%)	17 (8.0%)	17 (7.8%)	16 (6.8%)
**cyprus**	56 (8.4%)	11 (7.3%)	18 (6.9%)	27 (11%)	22 (10%)	18 (8.2%)	16 (6.8%)
**spain **	39 (5.9%)	2 (1.3%)	15 (5.7%)	22 (8.7%)	15 (7.1%)	15 (6.8%)	9 (3.8%)
**estonia**	215 (32%)	44 (29%)	87 (33%)	84 (33%)	50 (24%)	61 (28%)	104 (44%)
**germany **	93 (14%)	37 (25%)	29 (11%)	27 (11%)	29 (14%)	34 (16%)	30 (13%)
**hungary **	81 (12%)	19 (13%)	30 (11%)	32 (13%)	27 (13%)	23 (11%)	31 (13%)
**italy **	84 (13%)	22 (15%)	46 (18%)	16 (6.3%)	23 (11%)	40 (18%)	21 (8.9%)
**sweden**	48 (7.2%)	2 (1.3%)	14 (5.3%)	32 (13%)	29 (14%)	11 (5.0%)	8 (3.4%)
**ISCED**							
**low**	17 (2.7%)	9 (6.4%)	5 (2.0%)	3 (1.2%)	6 (2.9%)	6 (2.9%)	5 (2.2%)
**medium**	234 (37%)	59 (42%)	93 (37%)	82 (33%)	73 (36%)	76 (36%)	85 (37%)
**high**	389 (61%)	73 (52%)	152 (61%)	164 (66%)	125 (61%)	127 (61%)	137 (60%)
**BMI z-score**	0.38 (1.08)	0.45 (1.11)	0.46 (1.12)	0.27 (1.00)	0.28 (1.02)	0.44 (1.11)	0.43 (1.09)
**Waist z-score**	0.79 (1.20)	0.84 (1.24)	0.89 (1.21)	0.65 (1.15)	0.72 (1.15)	0.80 (1.21)	0.84 (1.23)
**BMI categories**							
**Normal weight**	526 (79%)	113 (75%)	202 (77%)	211 (83%)	177 (83%)	167 (76%)	182 (77%)
**Overweight**	40 (6.0%)	9 (6.0%)	22 (8.4%)	9 (3.5%)	8 (3.8%)	16 (7.3%)	16 (6.8%)
**Obesity **	100 (15%)	28 (19%)	38 (15%)	34 (13%)	27 (13%)	36 (16%)	37 (16%)
**DDS**	21.08 (5.12)	13.78 (2.72)	20.31 (1.60)	26.19 (1.88)	21.04 (5.39)	21.46 (4.60)	20.78 (5.33)
**DDS_Cereal**	4.50 (1.63)	3.77 (1.80)	4.27 (1.64)	5.16 (1.22)	4.27 (1.66)	4.47 (1.61)	4.72 (1.60)
**DDS_ Dairy**	4.57 (1.62)	3.29 (1.86)	4.48 (1.51)	5.43 (0.91)	4.80 (1.42)	4.87 (1.42)	4.09 (1.85)
**DDS_ Fruit**	3.60 (2.56)	1.14 (2.14)	3.27 (2.52)	5.39 (1.04)	3.49 (2.61)	3.60 (2.55)	3.70 (2.54)
**DDS_ Protein foods**	4.15 (1.57)	2.93 (1.53)	4.06 (1.46)	4.97 (1.15)	4.22 (1.59)	4.24 (1.46)	4.01 (1.64)
**DDS_ Vegetables**	4.26 (1.72)	2.65 (1.85)	4.24 (1.47)	5.24 (1.03)	4.25 (1.74)	4.28 (1.79)	4.26 (1.65)
**FVS**	0.68 (0.11)	0.69 (0.13)	0.68 (0.10)	0.68 (0.10)	0.55 (0.05)	0.67 (0.03)	0.80 (0.06)

Values are expressed as mean (SD) for continuous variables and as counts (%) for categorical variables. ISCED, International Standard Classification of Education. DDS, diet diversity score. FVS, food variety score.

**Table 5:** Characteristics of participants across DDS and FVS tertiles, age group ≥ 20 years.

≥20 Years	All	DDS			FVS		
		Low	Medium	High	Low	Medium	High
**No.**	1352	343	548	461	448	429	475
**Sex**							
**female**	940 (70%)	222 (65%)	376 (69%)	342 (74%)	286 (64%)	293 (68%)	361 (76%)
**male**	412 (30%)	121 (35%)	172 (31%)	119 (26%)	162 (36%)	136 (32%)	114 (24%)
**Age (years)**	42.4 (5.7)	42.8 (5.89)	42.4 (5.7)	42.1 (5.5)	43.1 (5.8)	42.2 (5.6)	42.0 (5.7)
**Countries**							
**belgium**	87 (6.4%)	25 (7.3%)	42 (7.7%)	20 (4.3%)	21 (4.7%)	37 (8.6%)	29 (6.1%)
**cyprus**	135 (10.0%)	42 (12%)	59 (11%)	34 (7.4%)	43 (9.6%)	50 (12%)	42 (8.8%)
**spain **	69 (5.1%)	8 (2.3%)	38 (6.9%)	23 (5.0%)	20 (4.5%)	36 (8.4%)	13 (2.7%)
**estonia**	562 (42%)	99 (29%)	209 (38%)	254 (55%)	186 (42%)	174 (41%)	202 (43%)
**germany **	193 (14%)	88 (26%)	75 (14%)	30 (6.5%)	65 (15%)	47 (11%)	81 (17%)
**hungary **	84 (6.2%)	42 (12%)	34 (6.2%)	8 (1.7%)	30 (6.7%)	11 (2.6%)	43 (9.1%)
**italy **	70 (5.2%)	27 (7.9%)	32 (5.8%)	11 (2.4%)	18 (4.0%)	32 (7.5%)	20 (4.2%)
**sweden**	152 (11%)	12 (3.5%)	59 (11%)	81 (18%)	65 (15%)	42 (9.8%)	45 (9.5%)
**ISCED**							
**low**	14 (1.1%)	8 (2.4%)	5 (0.9%)	1 (0.2%)	1 (0.2%)	7 (1.7%)	6 (1.3%)
**medium**	411 (31%)	140 (42%)	170 (32%)	101 (22%)	144 (33%)	121 (29%)	146 (31%)
**high**	897 (68%)	182 (55%)	363 (67%)	352 (78%)	290 (67%)	290 (69%)	317 (68%)
**BMI (kg/m^2^)**	25.54 (4.69)	26.18 (4.96)	25.79 (4.73)	24.76 (4.34)	25.41 (4.38)	25.68 (4.84)	25.54 (4.85)
**Waist (cm)**	85.43 (12.78)	87.41 (13.65)	85.89 (12.98)	83.46 (11.59)	85.79 (12.56)	85.85 (13.03)	84.70 (12.75)
**BMI categories**							
**Normal weight**	726 (54%)	164 (48%)	282 (51%)	280 (61%)	240 (54%)	226 (53%)	260 (55%)
**Overweight**	217 (16%)	71 (21%)	95 (17%)	51 (11%)	62 (14%)	77 (18%)	78 (16%)
**Obesity **	409 (30%)	108 (31%)	171 (31%)	130 (28%)	146 (33%)	126 (29%)	137 (29%)
**DDS**	20.54 (5.08)	13.68 (2.59)	20.29 (1.64)	25.93 (1.78)	20.36 (5.15)	20.95 (4.87)	20.33 (5.17)
**DDS_Cereal**	4.28 (1.49)	3.45 (1.42)	4.06 (1.43)	5.17 (1.10)	4.18 (1.48)	4.29 (1.53)	4.36 (1.46)
**DDS_ Dairy**	4.13 (1.80)	2.72 (1.74)	4.10 (1.66)	5.22 (1.16)	4.10 (1.77)	4.19 (1.74)	4.10 (1.88)
**DDS_ Fruit**	3.96 (2.16)	2.08 (2.18)	4.07 (1.97)	5.23 (1.15)	4.00 (2.16)	4.12 (2.11)	3.78 (2.20)
**DDS_ Protein foods**	4.02 (1.67)	2.92 (1.62)	3.94 (1.57)	4.94 (1.24)	4.04 (1.70)	4.12 (1.61)	3.91 (1.69)
**DDS_ Vegetables**	4.14 (1.74)	2.51 (1.36)	4.12 (1.59)	5.38 (0.99)	4.03 (1.78)	4.23 (1.69)	4.17 (1.74)
**FVS**	0.68 (0.11)	0.69 (0.12)	0.68 (0.11)	0.68 (0.09)	0.56 (0.05)	0.67 (0.03)	0.79 (0.06)

Values are expressed as mean (SD) for continuous variables and as counts (%) for categorical variables. ISCED, International Standard Classification of Education. DDS = diet diversity score. FVS = food variety score.

**Table 6:** Diet quality and nutritional content according to DDS and FVS tertiles and age groups.

		DDS			FVS	
	Low	Medium	High	Low	Medium	High
**6- < 12 yrs**						
**Energy (kcal/day)**	1499 ^mh^ (1407–1592)	1623 ^h^ (1536–1710)	1841 (1748–1934)	1718 ^mh^ (1626–1810)	1629 (1539–1720)	1565 (1470–1661)
**Protein (%TEI)**	16.4 (15.7–17.0)	16.6 (16.0–17.1)	16.5 (15.8–17.1)	16.5 (15.9–17.1)	16.6 (16.0–17.2)	16.3 (15.7–17.0)
**Total fat (%TEI)**	32.3 ^h^ (31.0–33.6)	32.6 ^h^ (31.3–33.8)	33.9 (32.6–35.2)	33.0 (31.7–34.3)	32.7 (31.5–34.0)	32.8 (31.5–34.2)
**SFA (%TEI)**	12.8 ^h^ (12.1–13.5)	13.2 (12.6–13.9)	13.8 (13.1–14.5)	13.6 ^h^ (12.9–14.3)	13.2 (12.5–13.9)	12.9 (12.2–13.6)
**Total carb (%TEI)**	51.2 ^h^ (49.8–52.7)	50.6 (49.2–52.0)	49.4 (47.9–50.8)	50.3 (48.9–51.7)	50.5 (49.1–51.8)	50.7 (49.2–52.1)
**Sugar (%TEI)**	18.9 (17.6–20.1)	19.7 (18.5–20.9)	19.8 (18.5–21.06)	19.2 (17.9–20.4)	19.4 (18.2–20.6)	19.8 (18.5–21.1)
**Fiber (g/day)**	12.2 ^mh^ (11.4–13.0)	14.1 ^h^ (13.4–14.8)	15.1 (14.4–15.9)	13.9 (13.1–14.6)	13.6 (12.8–14.3)	13.8 (13.0–14.6)
**UPFs (%TEI)**	50.2 ^h^ (46.0–54.4)	46.4 (42.5–50.3)	44.7 (40.5–48.9)	46.9 (42.8–50.9)	47.2 (43.2–51.2)	47.7 (43.5–51.9)
**Energy density**	1.02 ^mh^ (0.97–1.07)	0.95 (0.91–1)	0.96 (0.91–1.01)	0.99 (0.95–1.04)	0.98 (0.93–1.02)	0.96 (0.92–1.01)
**HDAS**	15.5 ^h^ (14.1–16.9)	16.07 (14.7–17.4)	17.2 (15.8–18.6)	16.2 (14.8–17.5)	16.0 (14.6–17.3)	16.5 (15.1–18.0)
**Meal freq. (time/day)**	4.50 ^mh^ (4.35–4.65)	4.66 (4.52–4.81)	4.73 (4.58–4.88)	4.61 (4.47–4.76)	4.68 (4.54–4.82)	4.57 (4.41–4.72)
**Junk food (time/day)**	1.73 (1.46–2)	1.58 (1.33–1.83)	1.54 (1.27–1.8)	1.53 ^h^ (1.27–1.79)	1.59 (1.34–1.85)	1.77 (1.5–2.03)
**FV (time/day)**	2.19 ^mh^ (1.86–2.53)	2.70 ^h^ (2.38–3.02)	3.11 (2.77–3.45)	2.49 (2.16–2.82)	2.74 (2.41–3.06)	2.68 (2.34–3.03)
**Fiber-rich foods (time/day)**	2.80 ^mh^ (2.33–3.27)	3.53 ^h^ (3.09–3.98)	3.92 (3.44–4.39)	3.28 (2.82–3.74)	3.47 (3.01–3.92)	3.39 (2.9–3.87)
**10 < 20 yrs**						
**Energy (kcal/day)**	1394 ^mh^ (1258–1530)	1634 ^h^ (1508–1761)	1921 (1797–2045)	1783 ^h^ (1651–1915)	1683 ^h^ (1547–1819)	1533 (1397–1669)
**Protein (%TEI)**	16.4 (15.5–17.4)	16.2 (15.3–17.05)	16.2 (15.3–17.0)	16.4 (15.5–17.2)	16.2 (15.3–17.1)	16.1 (15.2–17.0)
**Total fat (%TEI)**	30.1 ^mh^ (28.3–31.9)	31.9 ^h^ (30.1–33.6)	33.4 (31.7–35.1)	32.3 (30.6–34.0)	32.3 (30.6–34.1)	31.1 (29.3–32.8)
**SFA (%TEI)**	12.4 ^h^ (11.5–13.4)	13.0 (12.1–13.9)	13.7 (12.8–14.6)	13.5 ^h^ (12.6–14.4)	13.3 ^h^ (12.4–14.2)	12.3 (11.4–13.3)
**Total carb (%TEI)**	53.2 ^h^ (51.2–55.3)	51.7 (49.8–53.6)	50.2 (48.3–52.1)	51.0 (49.1–52.9)	51.3 (49.4–53.3)	52.5 (50.5–54.4)
**Sugar (%TEI)**	18.1 (16.3–19.8)	18.9 (17.2–20.5)	19.5 (17.9–21.1)	18.6 (17.0–20.3)	19.3 (17.7–21.0)	18.8 (17.1–20.4)
**Fiber (g/day)**	12.5 ^h^ (11.2–13.7)	13.5 ^h^ (12.4–14.6)	14.9 (13.7–16.0)	13.9 (12.8–15.1)	13.7(12.6–14.9)	13.5 (12.3–14.6)
**UPFs (%TEI)**	54.1 ^h^ (48.1–60.1)	48.6 (43.0–54.2)	47.0 (41.6–52.5)	47.2 (41.7–52.7)	50.8 (45.1–56.4)	51.4 (45.7–57.1)
**Energy density**	0.99 (0.92–1.06)	0.94 (0.88–1.01)	0.95 (0.89–1.01)	0.97 (0.91–1.04)	0.96 (0.89–1.02)	0.94 (0.88–1)
**HDAS**	16.1 ^mh^ (14.1–18.2)	18.0 (16.1–19.9)	19.1 (17.2–21.0)	17.8 (15.9–19.7)	18.5 (16.5–20.5)	17.5 (15.5–19.4)
**Meal freq. (time/day)**	3.78 ^mh^ (3.53–4.04)	4.14 (3.9–4.37)	4.13 (3.9–4.37)	4.11 (3.87–4.34)	4.04 (3.8–4.29)	3.93 (3.68–4.17)
**Junk food (time/day)**	1.99 (1.41–2.57)	1.91 (1.38–2.43)	1.74 (1.22–2.27)	1.95 (1.42–2.48)	1.77 (1.23–2.3)	1.87 (1.32–2.41)
**FV (time/day)**	2.55 ^mh^ (1.79–3.31)	3.36 (2.66–4.06)	3.75 (3.06–4.44)	3.29 (2.6–3.99)	3.58 (2.86–4.3)	2.96 (2.23–3.69)
**Fiber-rich foods (time/day)**	3.13 ^mh^ (2.13–4.13)	3.98 (3.06–4.9)	4.61 (3.71–5.51)	4.14 (3.22–5.06)	4.23 (3.28–5.18)	3.66 (2.71–4.61)
**≥20 yrs**						
**Energy (kcal/day)**	1502 ^mh^ (1388–1617)	1781 ^h^ (1670–1892)	2006 (1889–2124)	1842 ^mh^ (1720–1964)	1743 ^h^ (1624–1862)	1629 (1510–1748)
**Protein (%TEI)**	18.2 (17.3–19.1)	18.2 (17.3–19.0)	17.9 (17.0–18.8)	17.9 (17.1–18.8)	18.1 (17.3–19.0)	18.2 (17.4–19.1)
**Total fat (%TEI)**	34.7 ^mh^ (33.2–36.3)	36.0 (34.5–37.5)	36.4 (34.8–38.0)	35.7 (34.2–37.3)	35.4 (33.9–36.9)	35.7 (34.2–37.2)
**SFA (%TEI)**	13.5 (12.6–14.4)	14.0 (13.1–14.9)	14.1 (13.2–15.0)	14.0 (13.1–14.9)	13.8 (12.9–14.7)	13.8 (12.9–14.6)
**Total carb (%TEI)**	44.2 (42.4–46.0)	43.7 (42.0–45.4)	44.2 (42.4–46.0)	44.6 ^h^ (42.8–46.4)	44.2 (42.4–45.9)	43.4 (41.7–45.1)
**Sugar (%TEI)**	14.2 ^mh^ (12.8–15.6)	15.3 (14.0–16.7)	16.1 (14.6–17.5)	15.1 (13.6–16.5)	15.1 (13.7–16.5)	15.1 (13.7–16.5)
**Fiber (g/day)**	14.3 ^mh^ (13.0–15.5)	16.8 ^h^ (15.6–18.0)	18.0 (16.7–19.3)	17.3 ^mh^ (16.0–18.6)	16.2 ^h^ (14.9–17.4)	15.3 (14.0–16.5)
**UPFs (%TEI)**	42.6 (37.4–47.8)	40.3 (35.3–45.4)	40.1 (34.8–45.4)	39.3 (34.1–44.5)	41.3 (36.2–46.4)	42.2 (37.1–47.3)
**Energy density**	0.77 (0.72–0.83)	0.77 (0.72–0.82)	0.77 (0.72–0.83)	0.78 (0.72–0.83)	0.77 (0.71–0.82)	0.77 (0.72–0.83)
**HDAS**	24.1 ^mh^ (22.0–26.1)	25.6 (23.6–27.6)	26.6 (24.5–28.7)	25.8 (23.8–27.9)	25.2 (23.2–27.2)	24.9 (22.8–26.9)
**Meal freq. (time/day)**	2.89 ^mh^ (2.77–3.02)	3.1 ^h^ (2.98–3.23)	3.27 (3.14–3.4)	3.15 ^mh^ (3.02–3.28)	3.07 (2.94–3.2)	2.99 (2.86–3.12)
**Junk food (time/day)**	1.05(0.8–1.29)	1.01 (0.77–1.26)	1.05 (0.79–1.3)	1.01 (0.76–1.26)	1.06 (0.82–1.3)	1.03 (0.78–1.27)
**FV (time/day)**	2.36 ^mh^ (1.98–2.74)	2.95 (2.58–3.32)	3.1 (2.71–3.49)	2.77 (2.38–3.16)	2.78 (2.41–3.16)	2.73 (2.34–3.11)
**Fiber-rich foods (time/day)**	3.15 ^mh^ (2.58–3.72)	3.98 ^h^(3.43–4.54)	4.44 (3.86–5.02)	4.05 ^h^ (3.47–4.63)	3.80 (3.24–4.37)	3.58 (3.01–4.15)

Values are expressed as means (95% CI). TEI, total daily energy intake; SFA, saturated fatty acid; carb, carbohydrates; UPFs, ultra-processed foods; HDAS, healthy dietary adherence score. Meal freq., meal frequency; FV, fruit and vegetable consumption. For each variable, superscript different lowercase letters in the same row indicate significant differences among tertiles: ^m^ = significant difference vs. medium; ^h^ = significant difference vs. high. Analysis adjusted for sex, age, country, family ISCED (International Standard Classification of Education), and BMI (body mass index). DDS, diet diversity score. FVS, food variety score.

**Table 7:** Health status according to DDS and FVS tertiles and age groups.

		DDS			FVS	
	Low	Medium	High	Low	Medium	High
**6- < 12 yrs**						
**BMI z-score**	0.41 (0.2–0.62)	0.50 (0.3–0.71)	0.56 (0.35–0.78)	0.44 (0.24–0.65)	0.45 (0.25–0.65)	0.59 (0.38–0.81)
**Waist z-score**	0.68 (0.55–0.8)	0.63 (0.51–0.74)	0.58 (0.46–0.71)	0.69 (0.57–0.81)	0.6 (0.49–0.72)	0.59 (0.47–0.72)
**SBP**	103.7 (102.0–105.4)	103.8 (102.2–105.3)	104.5 (102.8–106.3)	104.2 (102.57–105.82)	103.7 (102.1–105.3)	104.1 (102.4–105.8)
**DBP**	63.4 (62.2–64.6)	63.3 (62.1–64.4)	63.8 (62.5–65.0)	63.6 (62.5–64.8)	63.14 (62.0–64.3)	63.6 (62.4–64.8)
**HOMA-IR**	1.29 (1.03–1.56)	1.47 (1.23–1.71)	1.33 (1.06–1.6)	1.41 (1.16–1.65)	1.41 (1.16–1.66)	1.29 (1.02–1.55)
**HbA1c (%)**	5.01 (4.94–5.08)	5.00 (4.94–5.06)	5.00 (4.93–5.07)	5.02 (4.96–5.09)	5.00 (4.93–5.06)	4.99 (4.93–5.06)
**Glycaemia (mg/dL)**	93.2 (91.3–95.0)	93.2 (91.5–95.0)	93.8 (91.9–95.7)	93.9 (92.1–95.7)	93.0 (91.3–94.8)	93.1 (91.3–95.0)
**Triglycerides (mg/dL)**	65.6 (58.2–73.1)	67.0 (60.0–74.0)	65.0 (57.3–72.7)	68.4 (61.2–75.6)	63.6 (56.4–70.7)	66.0 (58.5–73.5)
**HDL (mg/dL)**	60.7 (57.7–63.8)	59.9 (57.0–62.8)	59.8 (56.6–63.0)	60.4 (57.4–63.4)	59.9 (56.9–62.9)	60.3 (57.1–63.4)
**LDL_(mg/dL)**	92.5 (87.1–97.9)	95.1 (90.0–100.3)	93.9 (88.2–99.5)	94.5 (89.2–99.8)	92.7 (87.5–97.9)	94.5 (89.0–100.0)
**10 < 20 yrs**						
**BMI z-score**	0.68 (0.39–0.97)	0.82 (0.56–1.08)	0.75 (0.49–1.01)	0.69 (0.43–0.95)	0.78 (0.51–1.04)	0.80 (0.54–1.07)
**Waist z-score**	0.95 (0.78–1.12)	0.91 (0.76–1.07)	0.84 (0.69–0.99)	0.92 (0.77–1.07)	0.86 (0.70–1.01)	0.90 (0.75–1.06)
**SBP**	108.8 (106.2–111.4)	109.5 (107.1–111.9)	109.9 (107.5–112.3)	109.7 (107.3–112.0)	108.7 (106.3–111.1)	109.9 (107.5–112.3)
**DBP**	65.4 (63.6–67.2)	65.0 (63.3–66.6)	65.4 (63.8–67.0)	65.4 (63.8–67.1)	64.9 (63.3–66.6)	65.4 (63.7–67.0)
**HOMA-IR**	2.11 (1.07–3.16)	1.68 (0.78–2.59)	1.83 (0.88–2.78)	1.71 (0.80–2.62)	1.77 (0.84–2.71)	2.14 (1.16–3.11)
**HbA1c (%)**	5.02 (4.93–5.11)	5.01 (4.93–5.09)	5.01 (4.92–5.09)	5.03 (4.95–5.11)	4.99 (4.91–5.07)	5.02 (4.93–5.1)
**Glycaemia (mg/dL)**	95.7 (93.5–98.0)	95.5 (93.5–97.5)	95.2 (93.1–97.2)	95.1 (93.1–97.1)	95.7 (93.6–97.7)	95.6 (93.5–97.7)
**Triglycerides (mg/dL)**	78.2 (66.6–89.7)	73.9 (63.6–84.1)	73.6 (63.4–83.9)	75.1 (65.0–85.3)	75.9 (65.5–86.3)	73.7 (63.1–84.3)
**HDL (mg/dL)**	55.3 (51.5–59.2)	56.7 (53.3–60.1)	57.3 (53.9–60.7)	55.4 (52.1–58.8)	56.9 (53.5–60.4)	57.7 (54.1–61.2)
**LDL_(mg/dL)**	87.5 (79.3–95.7)	84.6 (77.4–91.9)	89.2 (81.9–96.4)	85.5 (78.3–92.8)	88.7 (81.3–96.1)	87.7 (80.2–95.3)
**≥20 yrs**						
**BMI (kg/m^2^)**	27.21 (26.09–28.33)	27.37 (26.3–28.45)	26.77 (25.62–27.92)	26.97 (25.86–28.09)	27.32 (26.23–28.4)	27.18 (26.09–28.27)
**Waist (cm)**	86.9 (85.5–88.3)	86.6 (85.2–88.0)	86.5 (85.1–88.0)	86.8 (85.3–88.2)	86.8 (85.4–88.2)	86.5 (85.2–87.9)
**SBP**	119.4 (116.4–122.3)	119.4 (116.5–122.2)	118.7 (115.7–121.8)	119.5 (116.6–122.5)	118.8 (115.9–121.7)	119.5 (116.6–122.3)
**DBP**	77.0 (75.0–79.0)	76.8 (74.8–78.7)	75.9 (73.9–78.0)	76.7 (74.7–78.7)	76.3 (74.3–78.3)	77.0 (75.0–79.0)
**HOMA-IR**	0.98 (−9.85–11.81)	0.62 (−10.38–11.63)	6.16 (−5.79–18.11)	1.99 (−8.72–12.71)	5.44 (−8.57–19.46)	6.05 (−15.95–28.05)
**HbA1c (%)**	5.21 (5.12–5.31)	5.15 (5.06–5.24)	5.20 (5.1–5.29)	5.18 (5.09–5.28)	5.18 (5.09–5.28)	5.18 (5.09–5.28)
**Glycaemia (mg/dL)**	108.3 (105.4–111.3)	108.2 (105.4–111.0)	108.1 (105.1–111.1)	107.6 (104.7–110.5)	108.4 (105.6–111.3)	108.4 (105.6–111.3)
**Triglycerides (mg/dL)**	132.9 ^h^ (118.5–147.4)	124.0 (110.2–137.8)	118.6 (103.8–133.3)	121.8 (107.4–136.3)	126.1 (112.2–140.1)	128.6 (114.6–142.7)
**HDL (mg/dL)**	54.0 (50.5–57.5)	54.2 (50.9–57.6)	55.0 (51.4–58.5)	55.2 (51.7–58.6)	54.3 (51.0–57.7)	53.7 (50.3–57.1)
**LDL_(mg/dL)**	129.7 (121.8–137.6)	130.3 (122.8–137.8)	130.1 (122.1–138.2)	129.1 (121.3–137.0)	131.0 (123.4–138.6)	129.7 (122.1–137.4)

Values are expressed as means (95% CI). For each variable, superscript different lowercase letters in the same row indicate significant differences among tertiles: ^h^ = significant difference vs. high. Analysis adjusted for sex, age, country, family ISCED (International Standard Classification of Education), and BMI (body mass index); N (6 < 12 yrs) = HOMA-IR 455; HbA1c 309; glycemia 309; triglycerides, HDL, LDL 288. N (10 < 20 yrs) = HOMA-IR 245; HbA1c 142; glycemia 148; triglycerides, HDL, LDL 139. N (≥20 yrs) = HOMA-IR 1336; HbA1c 152; glycemia 143; triglycerides, HDL, LDL 129. DDS, diet diversity score. FVS, food variety score.

## Data Availability

The data that support the findings of this study are available from IDEFICS (http://www.idefics.eu, accessed on 7 September 2023) and the I.Family study (http://www.ifamilystudy.eu 7 September 2023) but restrictions apply to the availability of these data, which were used under license for the current study, and so are not publicly available. Data are however available from the authors upon reasonable request and with permission of the IDEFICS consortium.
